# Design of robot grippers for binder jet products handling

**DOI:** 10.1038/s41598-024-56385-8

**Published:** 2024-03-08

**Authors:** MA Muktadir, Sun Yi, Amelia M. Elliott

**Affiliations:** 1 Department of Mechanical Engineering, North Carolina A&T State University, Greensboro, NC USA; 2 Oak Ridge National Laboratory, Oak Ridge, TN USA

**Keywords:** Robot grippers, End effector, Industrial robot, Manipulator finger, Additive manufacturing, Binder jet, Energy science and technology, Engineering

## Abstract

Dimension accuracy, damage minimization, and defect detection are essential in manufacturing processes, especially additive manufacturing. These types of challenges may arise either during the manufacture of a product or its use. The repeatability of the process is vital in additive manufacturing systems. However, human users may lose concentration and, thus, would be a great alternative as an assistant. Depending on the nature of work, a robot’s fingers might vary, for example, mechanical, electrical, vacuum, two-fingers, and three-fingers. In addition, the end effector plays a vital role in picking up an object in the advanced manufacturing process. However, inbuilt robotic fingers may not be appropriate in different production environments. In this research presented here considering metal binder jet additive manufacturing, the two-finger end- effectors are proposed design, analysis, and experiment to pick up an object after completing the production process from a specific location. The final designs were further printed by using a 3D metal printer and installed in the existing robotic systems. These new designs are used successfully to hold the object from the specific location by reducing the contact force that was not possible with the previously installed end effector's finger. In addition, a numerical study was conducted in order to compare the flowability of the geometric shape of finger's free areas.

## Introduction

The revolution of Industry 4.0 is currently underway around the world. A manufacturing technique, additive manufacturing (AM), is replacing many traditional manufacturing. Researchers and educators are shifting their focus from manual to automatic tasks. AM began almost 150 years ago with roots in topography and photosculpture^1^. These processes are enhanced by robotic grippers, which offer numerous advantages. Firstly, grippers facilitate automated handling and manipulation of components, reducing manual intervention and improving efficiency. A second benefit is that they ensure consistency and repeatability in picking up, placing, and manipulating objects, which is crucial to achieving accuracy and quality in additive manufacturing. Furthermore, robotic grippers can handle various shapes, sizes, and materials, an essential capability, where components with different geometries are manufactured. Likewise, considering the multiple technologies involved in additive manufacturing, such as fused deposition modeling (FDM), stereolithography (SLA), selective laser sintering (SLS), binder jetting (BJ), etc., robotic grippers can be tailored to meet the specific requirements of each printing method. Last but not least, robotic grippers are adaptable. Depending on the additive manufacturing setup, they can be customized and scaled to meet varying production volumes, making them versatile and applicable to different manufacturing environments. In this study, a new gripper have been design, print and tested for BJ AM process.

The most common materials used in AM are polymers, metals, ceramics, and composites. Several AM processes exist, including binder jet additive manufacturing (BJAM)^2^. An early 1990s MIT invention, BJAM, involves forming cross-sections of parts^3^. It involves selectively depositing a liquid binding agent onto powder particles to join them to form a layer. It is also possible to print 3D models with ceramics, polymers, and sands using this method^4,5^. It is a non-melting process and is primarily consolidated by sintering, so porosities may always occur, and their volume, size, and shape may differ among parts made from the same batch because there is no melting. After printing, the part must be cured, sintered, and annealed, so the parts are expected to have a coarse microstructure. Binder saturation affects the quality, dimensional accuracy, density, and mechanical properties of parts. A powder bed's density and particle size influence the amount of binder that deposits on a part. Generally, fine and irregular-shaped particles require a higher binder saturation due to their lower powder bed density. Moreover, cracking or non-uniform shrinkage may occur when there are localized variations in density. Among the challenges facing metal BJ is the high level of shrinkage in contradiction with full densification^6,7^.

In 3D printing, binder jetting holds particular promise because it can produce complex structures quickly while achieving isotropic properties. In terms of speed and resolution, binder jetting has several advantages over other forms of AM, especially when it comes to parts with no residual thermal stress and an isotropic microstructure and properties, which can be achieved with BJ^8^. A crucial stage of BJAM is picking up the printed products from the powder bed in a system where a robot is used to do the repeated work. This study considered only the issue of reducing the crack loss or material loss, as well as the issue with gripping in the fragile parts in the granular bed, has been considered. The existing robot finger shows that it fails to grip the object and breaks it while picking it up.

Grippers find extensive use in industrial settings, prompting the development of new designs tailored to specific industries. These designs aim to minimize production costs and prevent damage. For instance, one study introduced a variable structure pneumatic soft robot capable of gripping irregular objects. This innovation enables the robot to adjust to various object sizes through active expansion or contraction, thereby enhancing the success rate of grasping and minimizing potential damage to the objects held^9^.A properly designed gripper finger can improve overall system performance, reduce robot inaccuracy, and increase work. Poor finger designs can also damage expensive workpieces, resulting in a reduction in productivity and reliability^10^.

A variety of robots are available with different payloads and sizes to accomplish different tasks, such as welding, painting, and cutting. A special end-effector called a tool is required for these robots. Others are designed to execute general operations based on task and operation environment, such as assembly and pick and place. Robots and their environments are only interconnected by their end-effectors. As a result, a robot's overall performance depends heavily on its end-effector, so research in this area is critically important^11^. There are many different types of robot end-effectors. Grippers are one of the types with usually two or three fingers of one degree of freedom. Parallel jaw grippers are most used in industry and are typically actuated by pneumatic systems. Using a linear motion system, both gripper attachments are kept parallel and collinear as they move away from and toward each other^11^. This study developed a design like that can only move in parallel directions.

When automating, choosing, or designing the gripper is often one of the last problems to solve, which quickly leads to compromises or using a common gripper that meets the requirements. With so many grippers on the market these days, it can be hard to find a gripper that goes beyond just satisfying the requirements. When the gripper selection is compromised, the full potential of the robotic cell cannot be utilized. By selecting a better gripper, you could improve the duty cycle and reliability of the whole system, as well as reduce the cost^12,13^. In gripper design, multiple factors must be considered, including the objects to be manipulated and the loading and unloading conditions. Mechanics, geometric and physical properties, and mechanical properties are the main components affecting gripper design requirements. A few of these factors are the shape, size, weight, and location of the center of gravity, as well as the material. The shape and size of the manipulated parts define the gripper's size. Weight and center of gravity determine the minimum force required to manipulate the part and where the gripper should be in contact with it. Gripping force is determined by the part's surface and friction coefficient^13,14^.

With additive manufacturing (AM), gripper systems that are multifunctional, fast to manufacture, and customizable can be developed. In recent years, Human–Robot-Collaboration (HRC) has developed rapidly with the help of lightweight collaborative robots. A safe gripper system is an important prerequisite for HRC. By integrating several components into a complex component, the subsequent assembly effort can also be reduced. AM offers new freedom in product design. As a result, lightweight components can be produced. An efficient and lightweight gripper has been developed for a robot in a study^15^. The primary objective of this study is to grip an object from the BJ manufacturing system that could not be gripped previously.

It is possible to design lightweight components with high performance by distributing material according to the load-carrying path of the part. This combination can be achieved by using finite element-based topology optimization. In topology optimization, specific pre-defined requirements are needed. The area in space where material must be present, points where voids or no material must be present, and the loads applied to the part. Topology optimization finds the optimal material layout within the given design space, resulting in weight-competitive structures^16,17^.

New developments in commercial 3D printing systems (machines and materials) have enabled soft and unconventional parts fabrication. For example, unlike traditional manufacturing methods such as molding and soft lithography, AM is positioned to become the future manufacturing technology in robotics^18^. In another study, using AM, an actuator was developed that was inspired by the motion mechanism of worms and designed to imitate the movement of a human finger. Since this actuator's external covers can be adapted according to the requested task and due to its modular design makes it possible to use for a wide range of applications, including soft grippers for fruit grasping, industrial grippers, and medical exoskeletons for patients who have mobility difficulties^19^. In this study, two types of 3D printers were used to print and tested by existing robots.

It is demonstrated in a paper how a robotic gripper specifically designed to grasp and handle textiles and soft flexible layers can be miniaturized and improved through polymeric additive manufacturing. Robotic gripping devices for non-rigid items demonstrate how additive manufacturing allows for smart design solutions. FE analysis and mechanical testing have validated the component designs^20^. It is necessary for industrial robots to have grippers with unique designs, limited volumes, and the lowest cost in order to work on customized applications. Aside from allowing for better design space for customized limited volume components, FEA-assisted topology optimization has shown great promise. A study designed a mini-robot to lift a 100-g component for palletizing using topology optimization^21^. In this study, the conditions are different, so topology analysis was conducted to reduce the finger material thickness as it is one of the factors affecting finger movement.

This study based on considering BJ automated pickup systems, a new robotic finger has been developed to address new challenges. According to the literature review and best of the knowledge the authors found that both the challenges and the solution approach are novel, and there is no standardization by which the results can be compared.

## Materials and method

**Figure 1:**
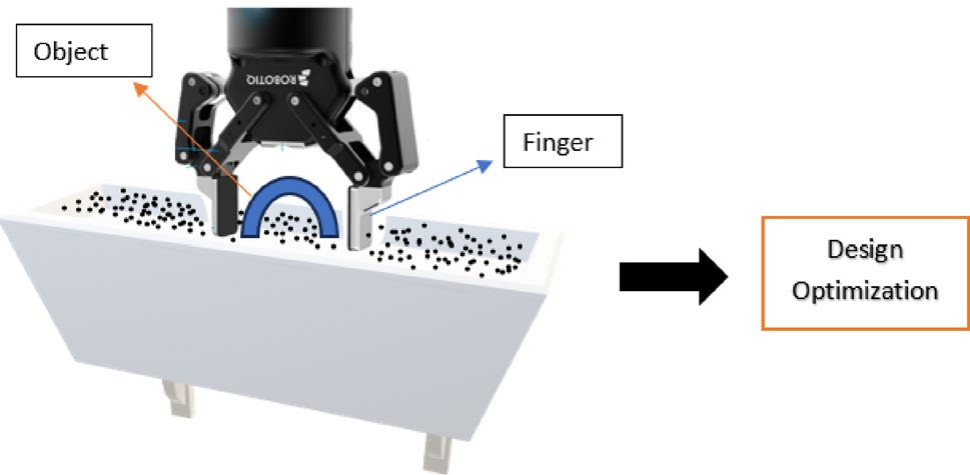
End effector (ROBOTIQ).

In binder jet printing, a liquid binding agent is dispensed onto the powder to create two-dimensional patterns. Stacking layers creates a physical object. In addition to printing techniques, powder deposition, and dynamic binder/powder interactions, Binder Jetting additive manufacturing (BJAM) can be applied to virtually any powder. Materials such as polymers, metals, and ceramics have been processed with BJAM. Additive manufacturing has different stages of production. To automate the process, the final products must be grasped by a robotic manipulator. This study demonstrates a special case of end effector grasps, where the design of the gripper was a must to grasp the product. The following Fig. 1 shows the schematic of the pick-up of an object after the BJ production, where the robotic finger has a vital effect as it is moving in a solid grain medium to touch from both sides of the target object, where the location is specific that the robot can control from the program by specifying the angle and x–y–z location.

**Figure 2:**
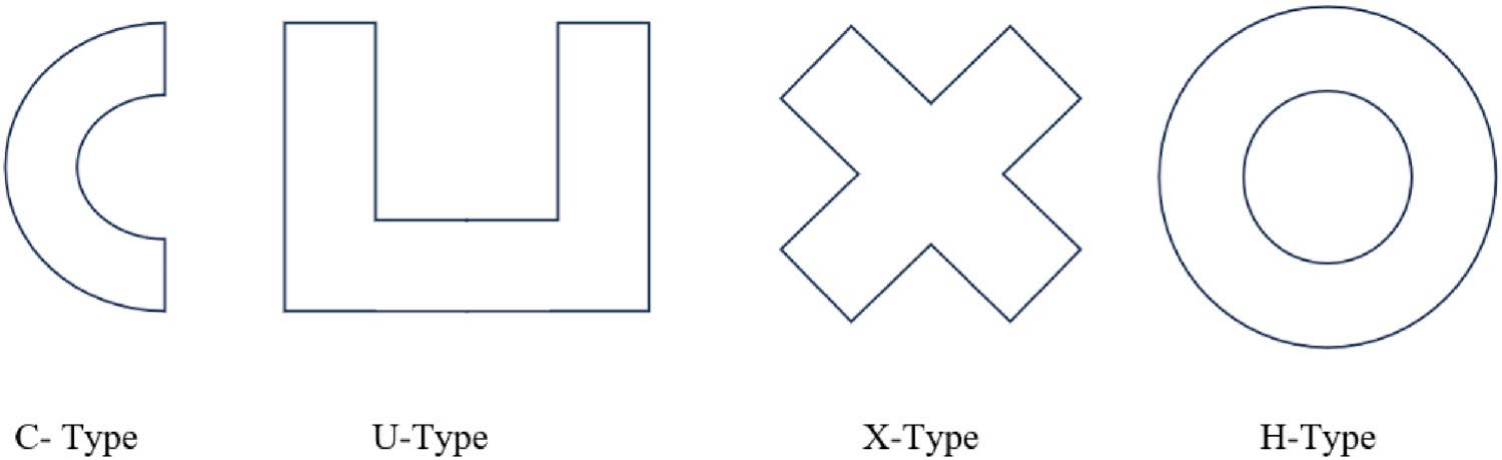
Types of models or samples.

**Figure 3:**
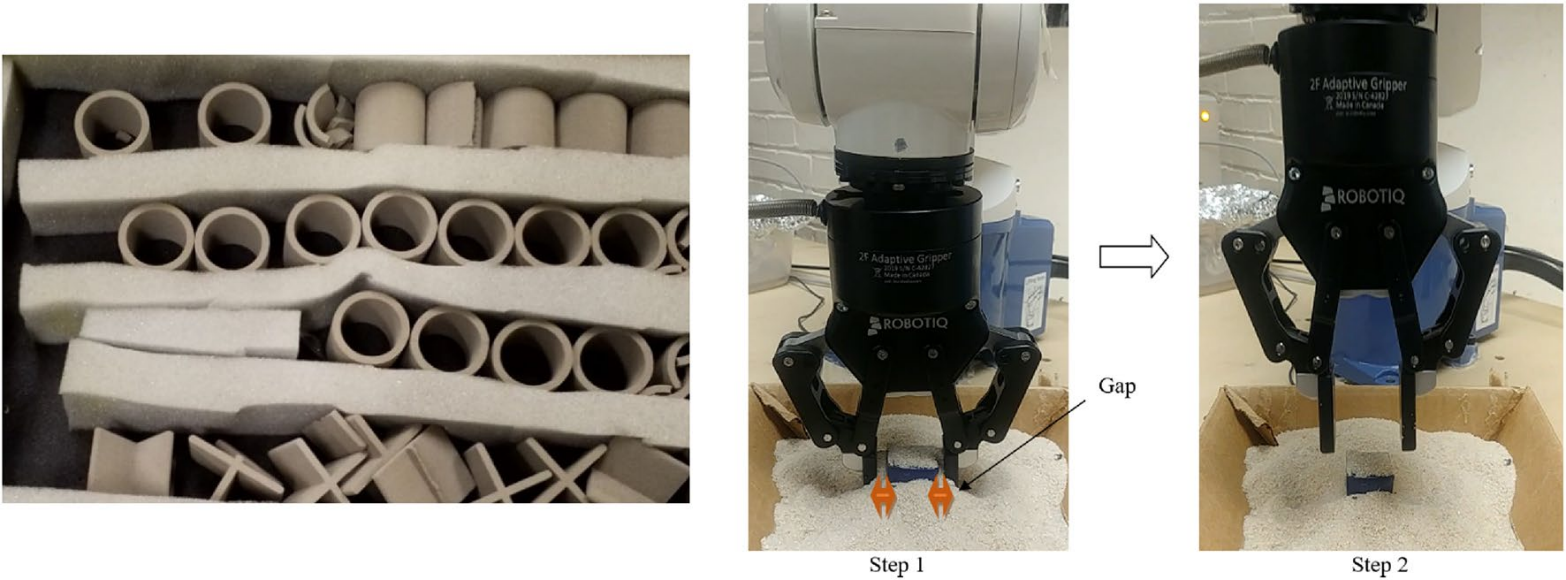
Original sample (left) and an experiment setup with an industrial robot (middle and right).

The following (Fig. 2) is the schematic of the model in a plane (x–y) of the three-dimensional model, and Fig. 3 shows the original sample of the BJ object.

**Figure 4:**
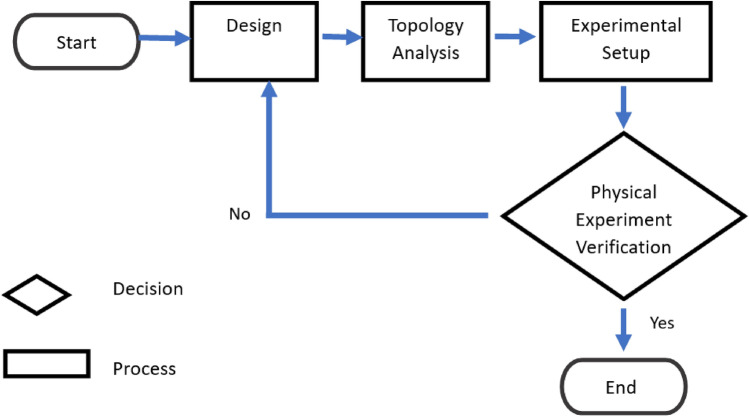
Flow chart of methodology.

**Figure 5:**
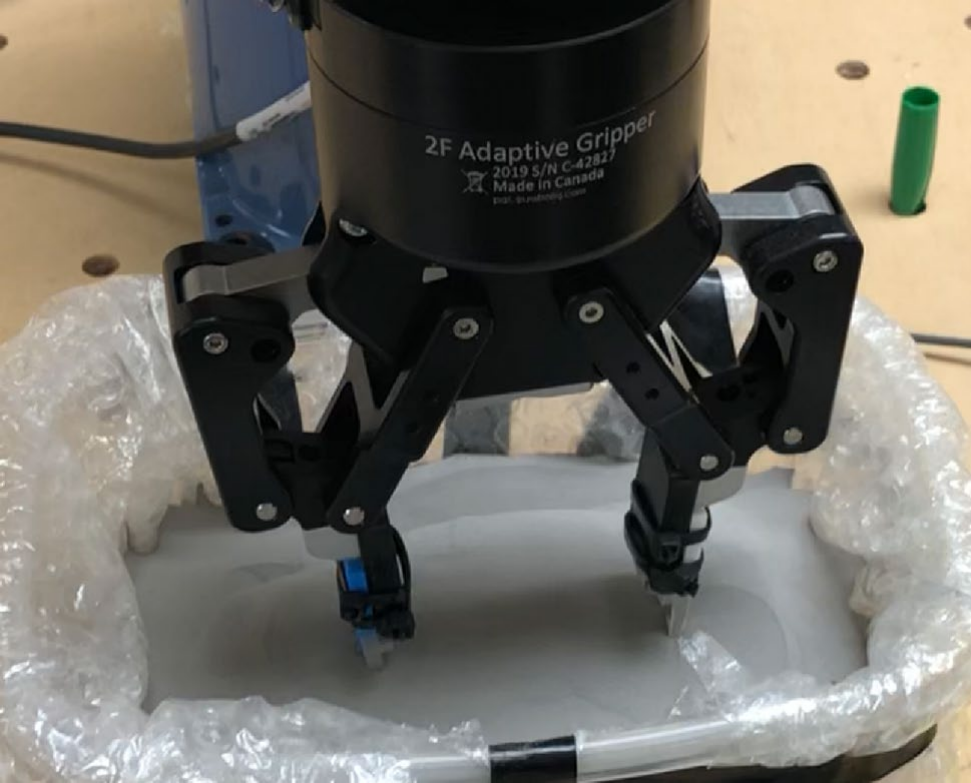
DENSO robot with the ROBOTIQ end effector.

Figure 3 (left) shows some broken parts due to the pick-up problem of the existing finger and an experiment shows that there has an gripping problem while to to pick it up (middle) and fails to gript (right). To solve that challenges this sudy desing, analyze and tested according to the following flowchart from the design to the experimental process, where SolidWorks is used for the design and topology analysis part (Fig. 4). For the experimental part, a Denso Robot with the ROBOTIQ end effector was used (Fig. 5).

For the experimental validation of the weak point of the model, an industrial robotic arm, Model VP-624 M, has been used with the ROBOTIQ, a 2-finger gripper (Fig. 5).

### Design

For the design process, two factors have been considered as the working medium (pick-up location) is metal powder instead of air. Firstly, the natural angle of repose of the finger wall. As decreases the wall thickness the natural inclination of the wall increases up to 90 degrees. In addition, according to Hagen, by 1852, he performed experiments with sand and discovered that the mass flow rate of granular particles was *m* ~ *ρg*^*1/2*^* D*^*5/2*^. Where ρ is the density of the particles, *g* is the acceleration, and* D* is the circular Orifice diameter^22,23^.

**Figure 6:**
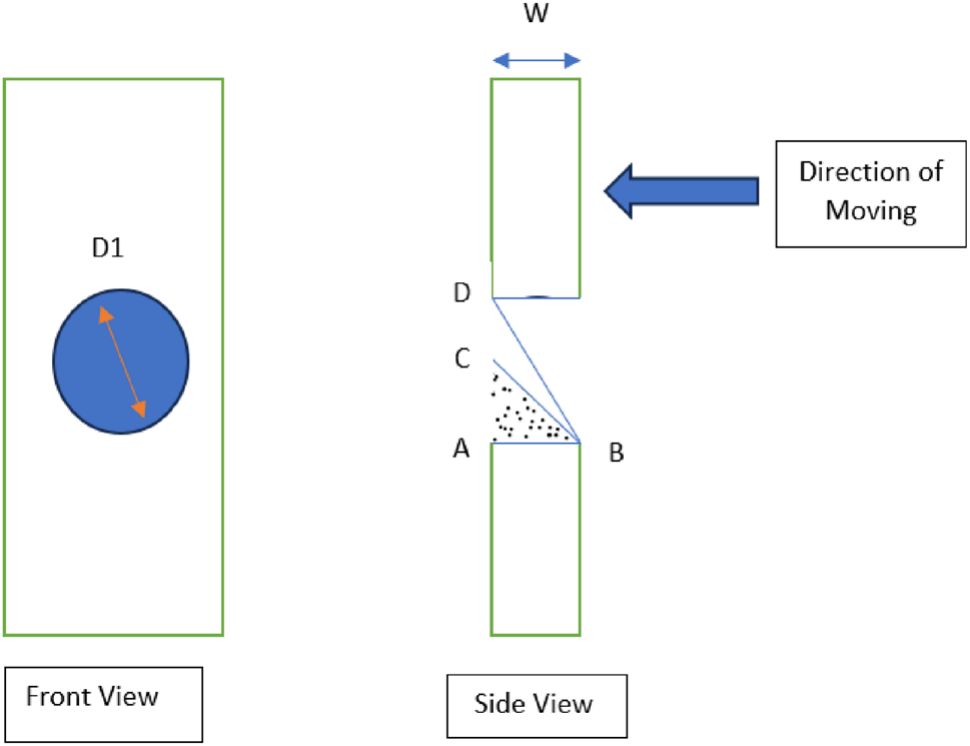
Front and side views of the fingers.

Following Fig. 6 shows a schematic diagram.

The angle of ABC = angle of repose.

The angle of ABD = natural inclination of the wall.

*D1* = diameter of the hole of the fingers.

Critical value = (*D*/tan (angle of repose)).

*W* = wall thickness.

Secondly, the ratio of the free area and the total area of the designed fingers. Which also affects granular flowability. Finally, topology optimization has also been done to reduce the surface area and wall thickness.

**Figure 7:**
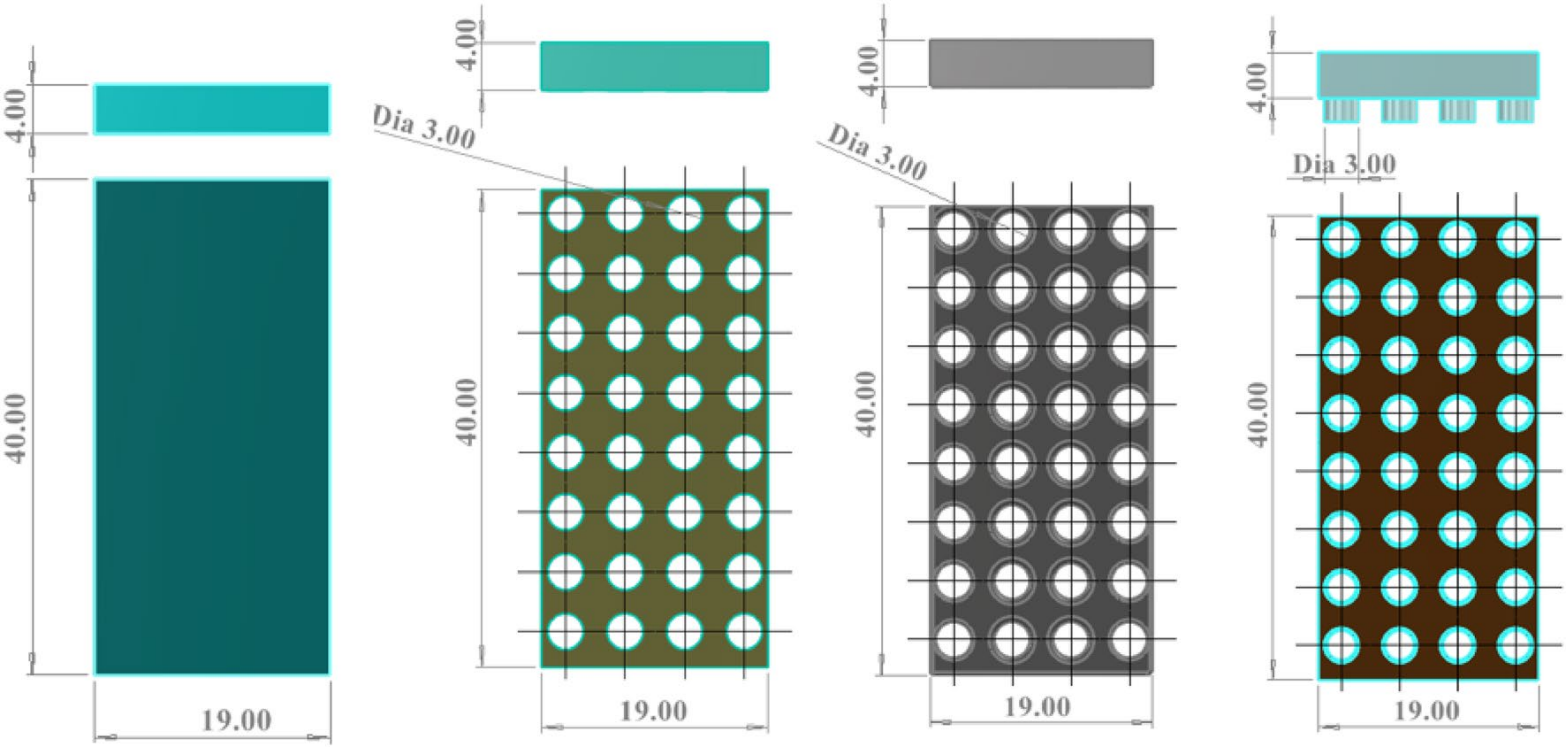
Drawing *of* V11, V12, V13, and V14 (left to right) where all dimensions are in 'mm'.

Figure 7 shows the preliminary design of Version (V) of 11, 12, 13, and 14, left to right, respectively. V11 is similar to the existing gripper fingers.

**Figure 8:**
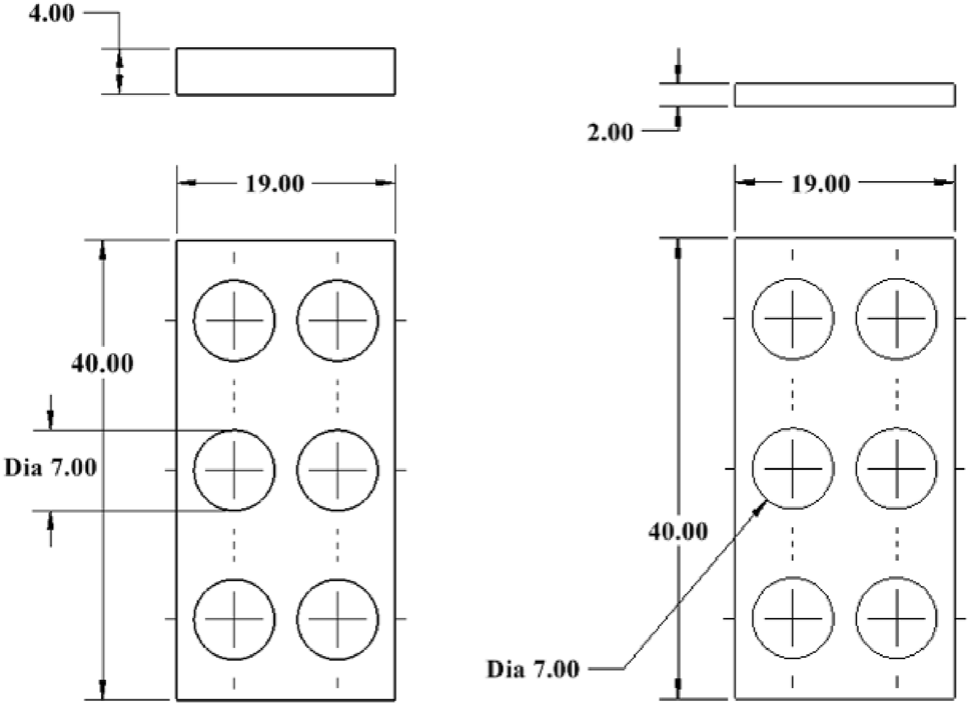
Drawing V13-A1 (left) and V13-A2 (right), where all dimensions are in 'mm'.

**Figure 9:**
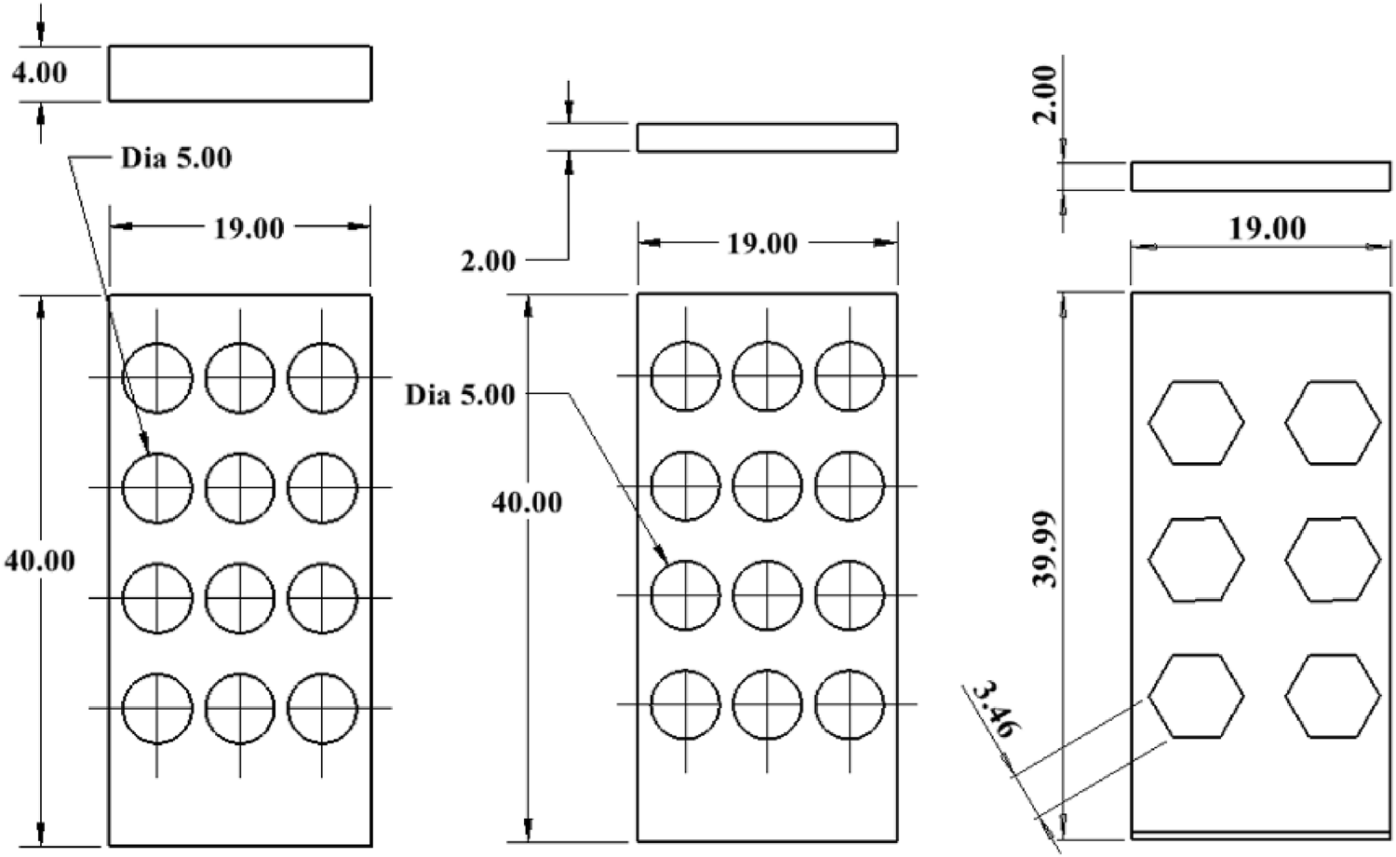
Drawing of V13-B1, V13-B2, and V13-B21 (left to right), where all dimensions are in 'mm'.

**Figure 10:**
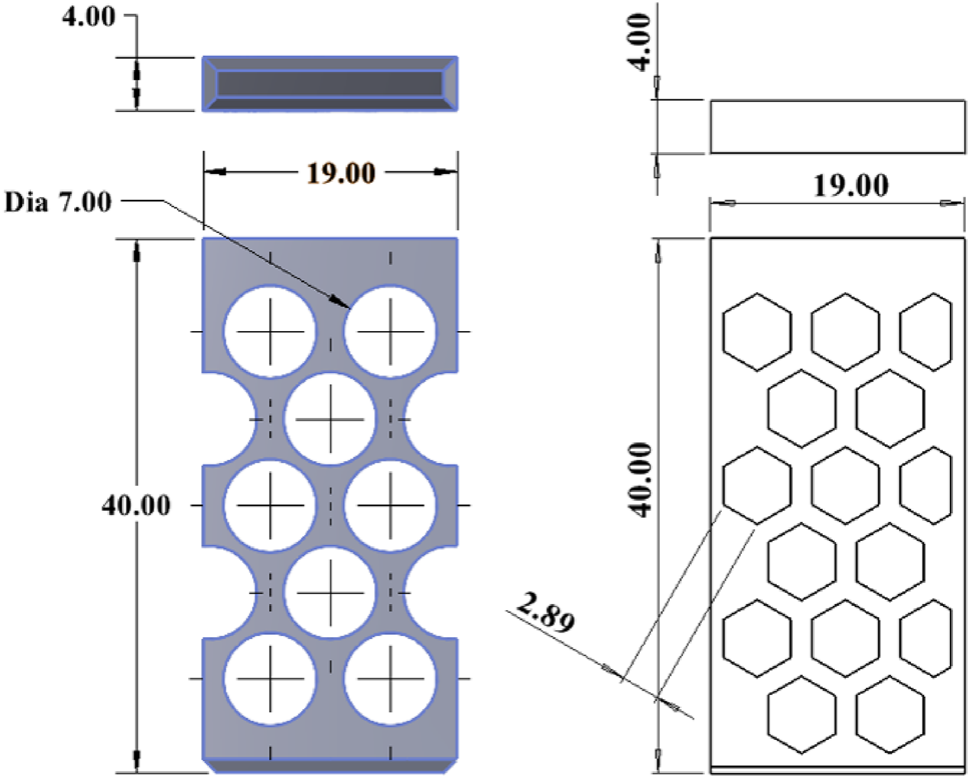
Drawing of V13-A11 and V13-B22.

Based on a preliminary simulation, design number V13 shows the lower normal force, and further design was modified based on that design. The hexagonal shape of the holes is also taken into consideration. Figures 8, 9, and 10 are the design of V13-A1, V13-A2, V13-B1, V13-B2, V13-B21, V13-A11, and V13-B22.

The following sections will analyze the designs of V13 to V13-B22 and topology analysis.

### Analyses of design

Table 1 compares the design model modified from design V13, where serial numbers 2 to 6 are circular, and serial numbers 7 and 8 have changed the hole shape to hexagonal. Columns G and H are the factors considered in that study to increase the flowability.

**Table 1:** Comparison of the designed finger of V13 to V13-B22.

Sl. no	Design number	Finger width (w) (mm)	Dia or length of the unit hole (mm)	Total area (mm × mm)	Free area (mm × mm)	Ratio %	The natural inclination of the wall (degree)
A	B	C	D	E	F	G = F/E	H
1	V13	4	3	760	226.20	29.76	36.86
2	V13-A1	4	7	760	230.91	30.38	60.26
3	**V13-A11**	4	7	760	384.85	**50.64**	**60.26**
4	V13-A2	2	7	760	230.91	30.38	74.05
5	V13-B1	4	5	760	235.62	31.00	51.34
6	V13-B2	2	5	760	235.62	31.00	68.19
7	V13-B21	2	3.46*	760	186.61	24.55	73.87
8	**V13-B22**	4	2.89*	760	303.80	**39.97**	**55.32**

*sl no. 7 and 8, column D is the one side length of a hexagonal hole.

Significant values are in bold.

**Figure 11:**
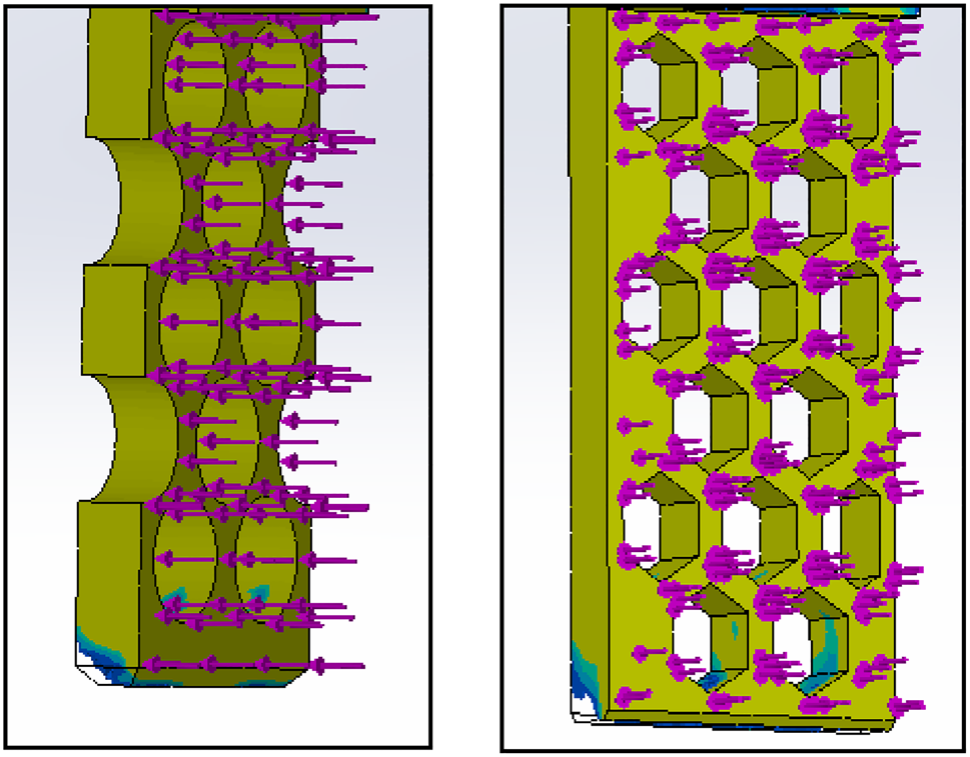
Topology analysis of V13-A11 and V13-B22 (left to right).

Based on the ratio (column G) and natural inclination (column H) of the wall, V13-A11 (circular) and V13-B22 (hexagonal) were considered for further design, topology analysis, and physical experimentation. Figure 11 shows the topology study of the V13-A11 and V13-B22, where both cases indicate that the fingers cannot be reduced in width.

**Figure 12:**
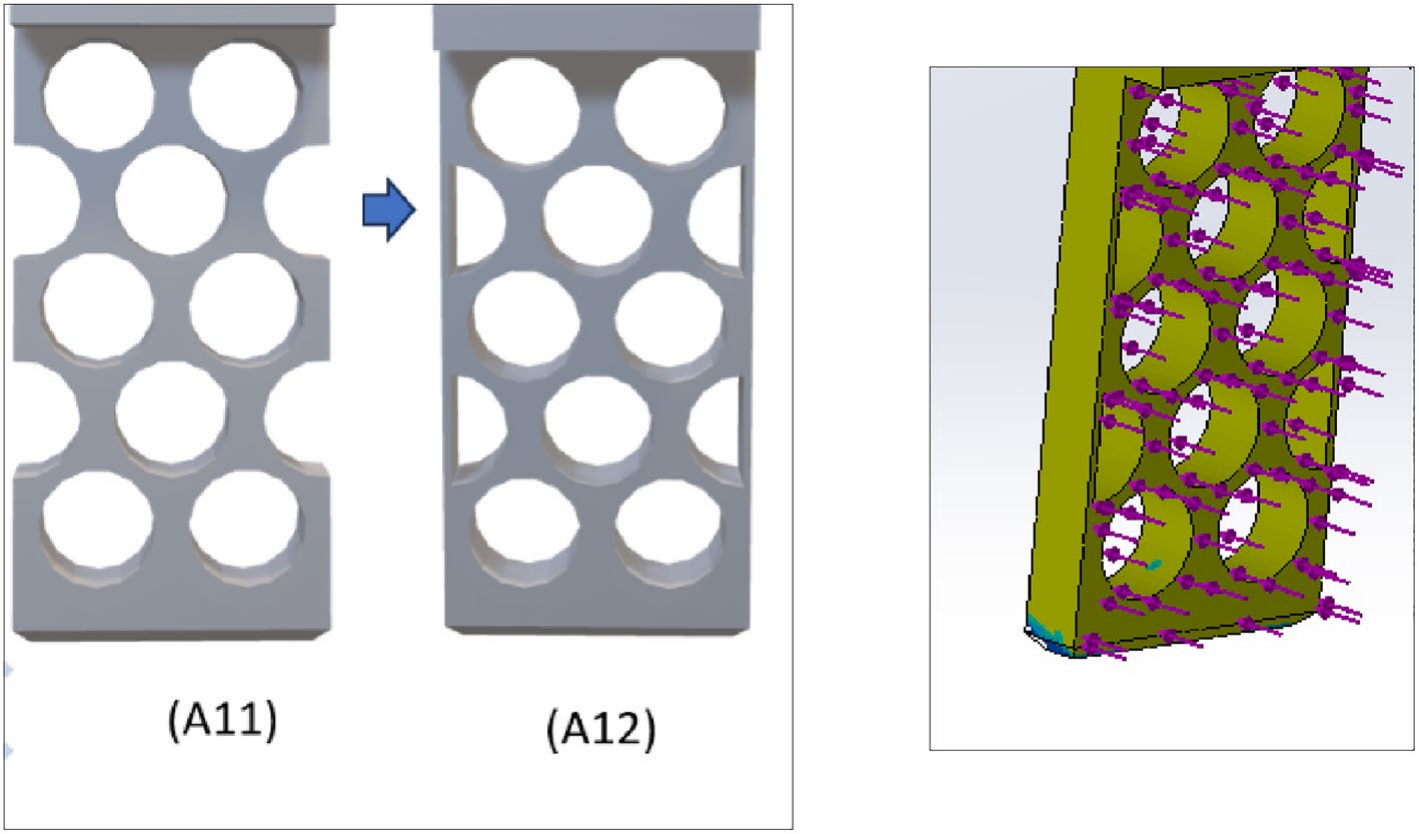
2D view of V13-A12 (left modified from V13-A11) and topology analysis.

**Figure 13:**
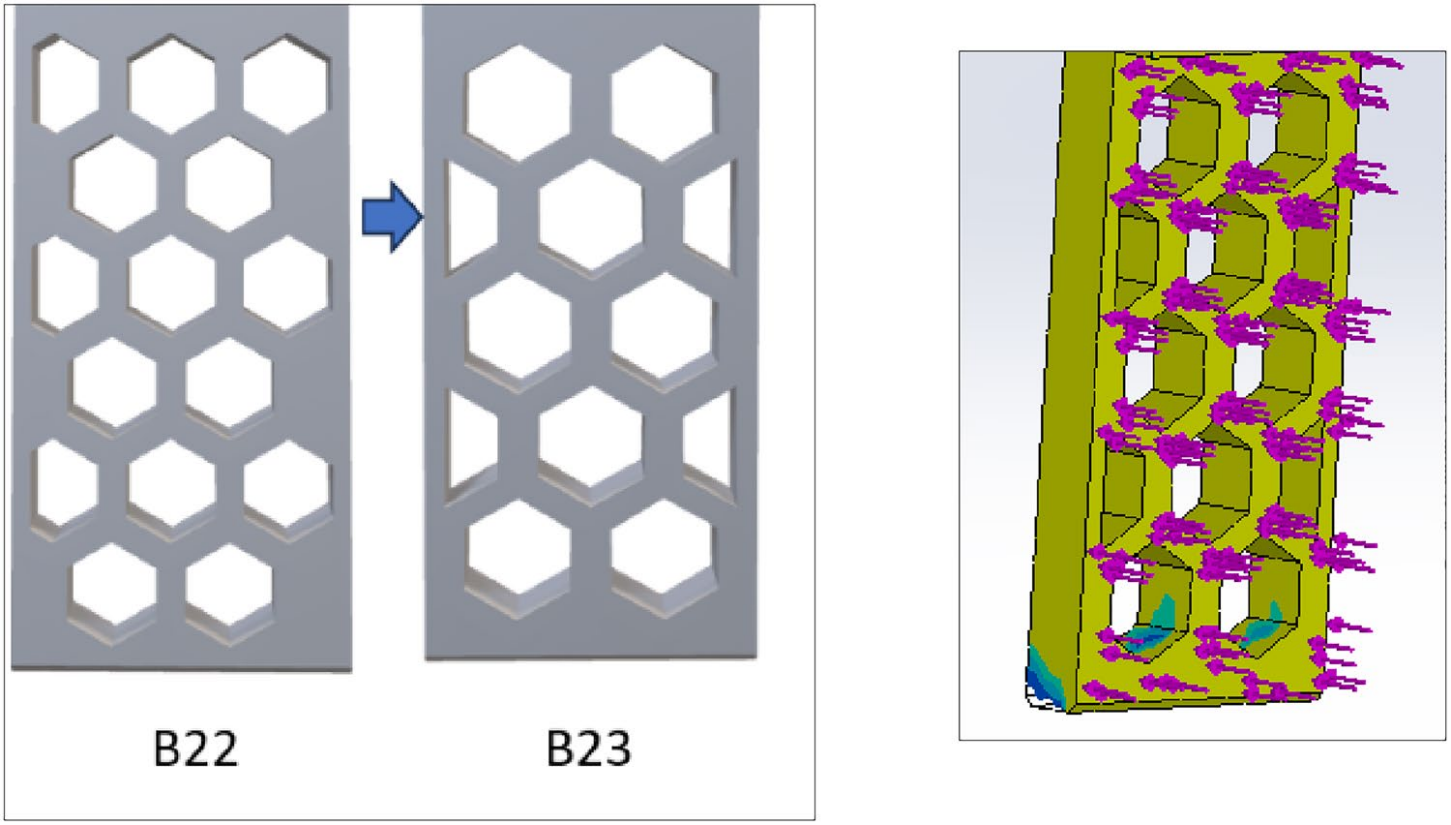
2D view of V13-B23 (left and modified from V13-B22) and topology analysis.

Figures 12 and 13 are the design and topology studies of models V13-A12 and V13-B23. V13-A12 is modified to reduce the corner sharpness that may break the object during pick-up from the BJAM. V13-B23 also has a slight difference. In that case, both the ratio and natural inclination have increased, increasing the granular flowability (Table 2). Per topology analysis, a thickness of less than 4 mm is not recommended with the existing material chosen for the printing and physical experiments.

**Table 2:** Comparison with the modified design.

Sl. no	Design number	Finger width (w) (mm)	Dia or length of the unit hole (mm)	Total area (mm × mm)	Free area (mm × mm)	Ratio %	The natural inclination of the wall (degree)
A	B	C	D	E	F	G = F/E	H
1	V13-A11	4	7	760	384.85	50.64	60.26
2	V13-A12	4	7	760	384.85	50.64	60.26
3	V13-B22	4	2.89*	760	303.80	39.97	55.32
4	V13-B23	4	3.5*	760	318.26	**41.88**	**60.25**

*sl no. 3 and 4, column D is the one side length of a hexagonal hole.

Significant values are in bold.

### 3D print and experimental test

**Figure 14:**
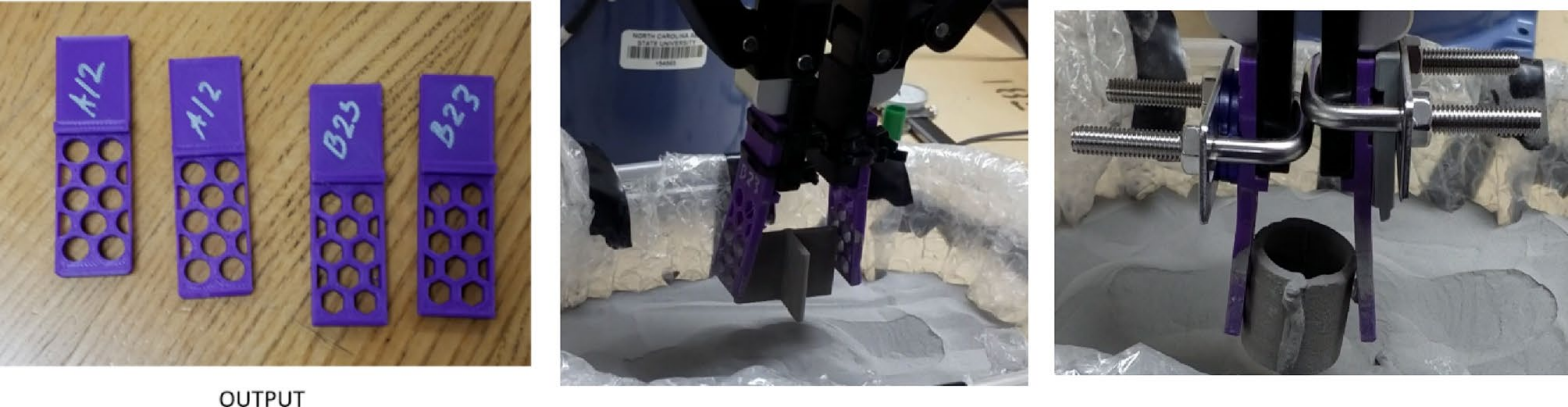
The left picture shows the printed fingers of versions A12 and B23 (where the upper part is only for the connection of the existing gripper). The middle and right pictures show the pick-up of an object from the metal particle.

Figure 14 shows (left) the fingers after printing with a conventional 3D printer and materials installed in the robot for testing. Additionally, the experiment indicates that both designs can grip the granular medium. However, there is a problem with the fingers bending (Fig. 14, middle and right). In the experiment, the powder used has a diameter of 15–20 µm, density of 7990 kg/m^3^, Young's modulus of 190 GPA, and Poisson's ratio of 0.265. For printing, PLA (Polylactic acid) was used as a material on a traditional 3D printer (CREALITY). Stainless steel Laser Form 17-4PH (A) is used as the material for metal printing on a 3D printer of the model ProX-200 DMP -3D.

**Figure 15:**
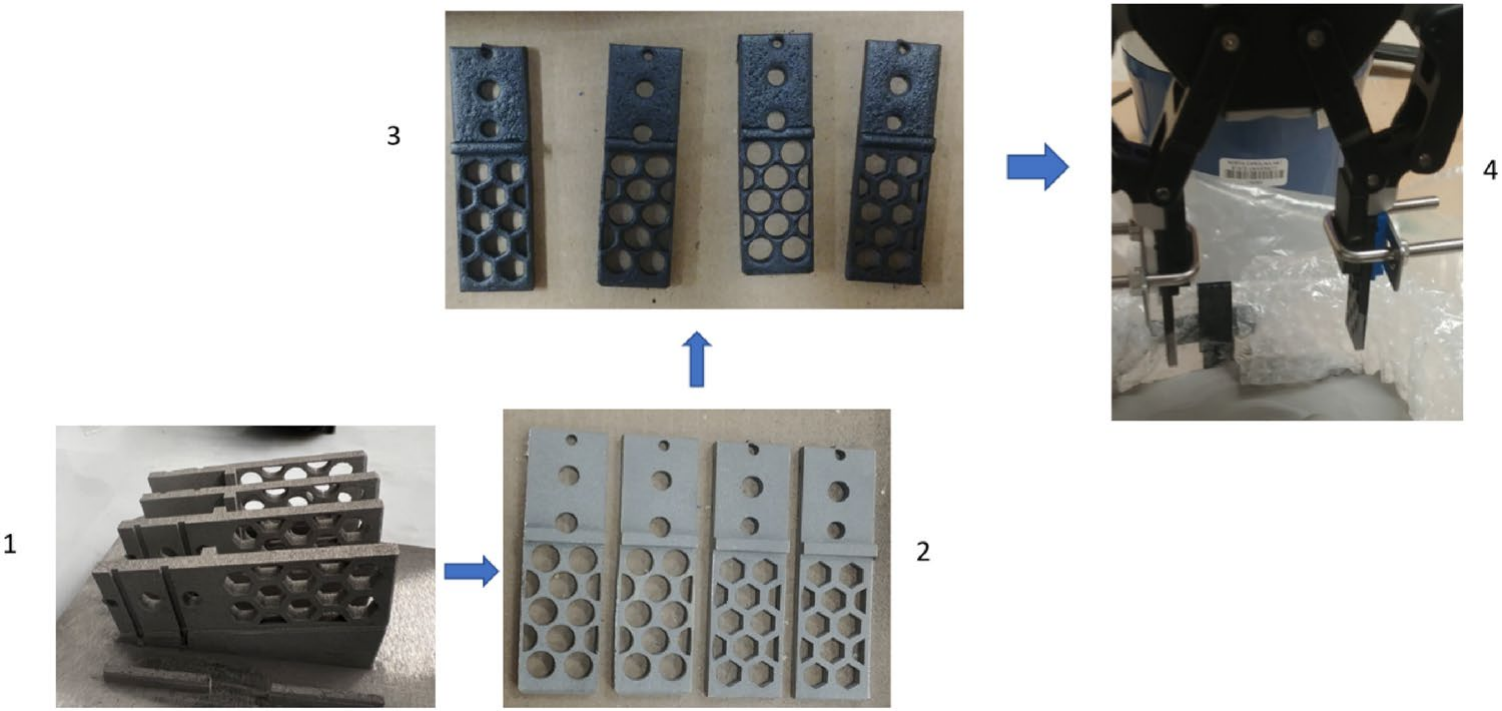
Metal printing steps and experimental setup.

**Figure 16:**
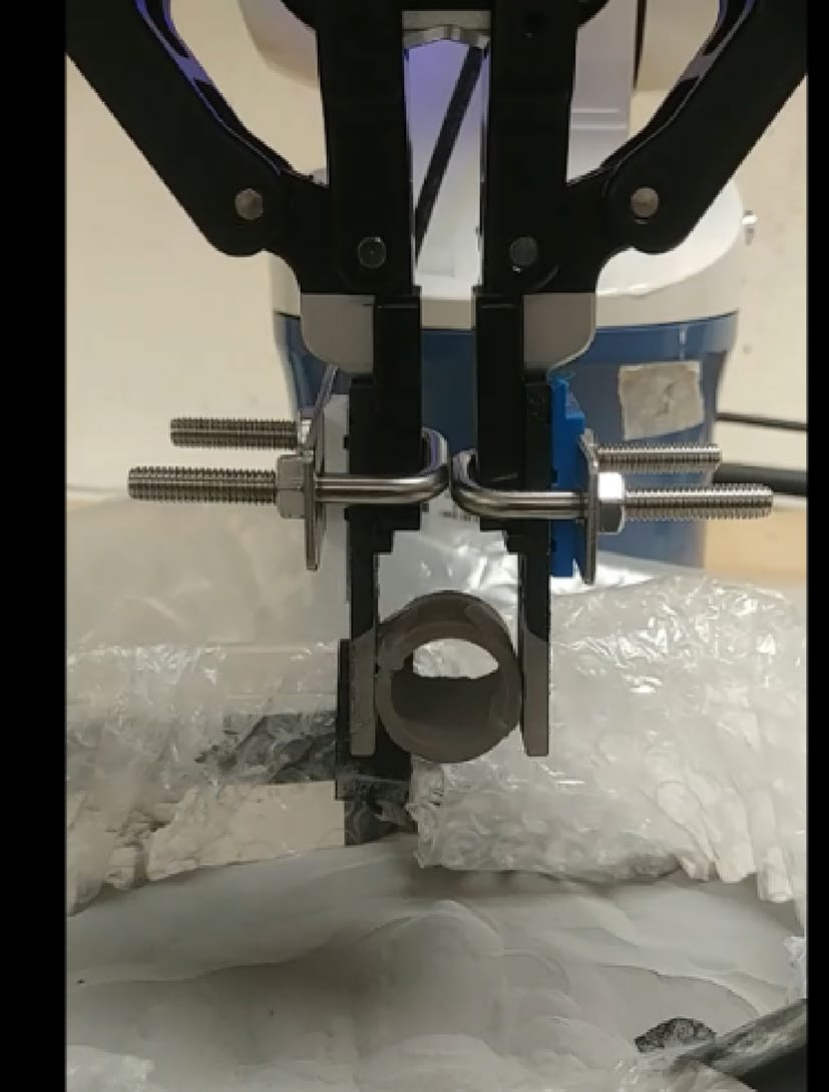
Experiment with an H-shaped object.

Printing the fingers with a metal additive manufacturing shows the solution to the bending issues of the previous experiments. Figure 15, a new experimental setup after printing, where a special coating is applied before installation to reduce the sharpness of the metal surface. It is evident from the experimental results that the problem is solved (Fig. 16).

## Results

**Figure 17:**
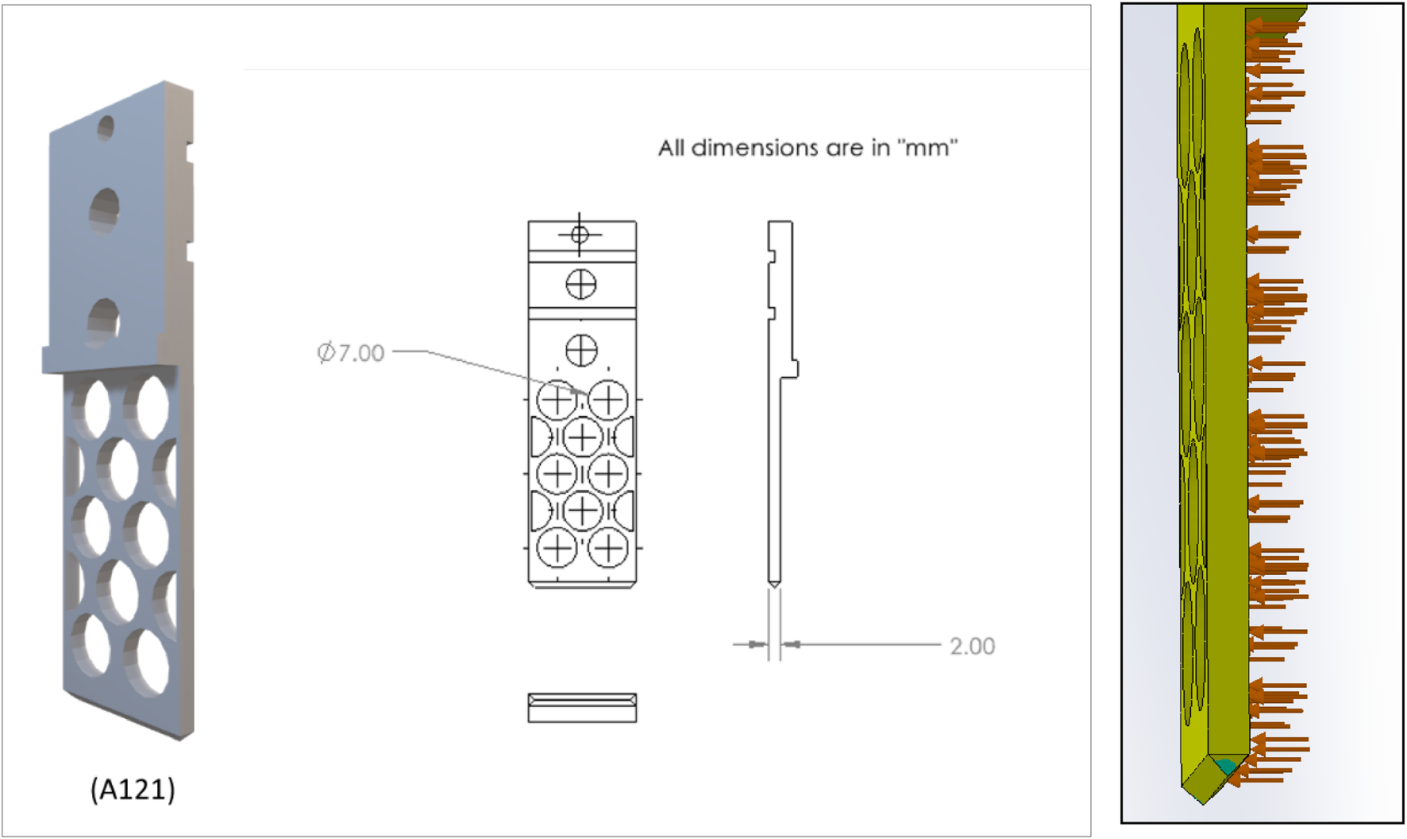
Design and 3D view of V13-A121 and topology study of V13-A121 (right).

**Figure 18:**
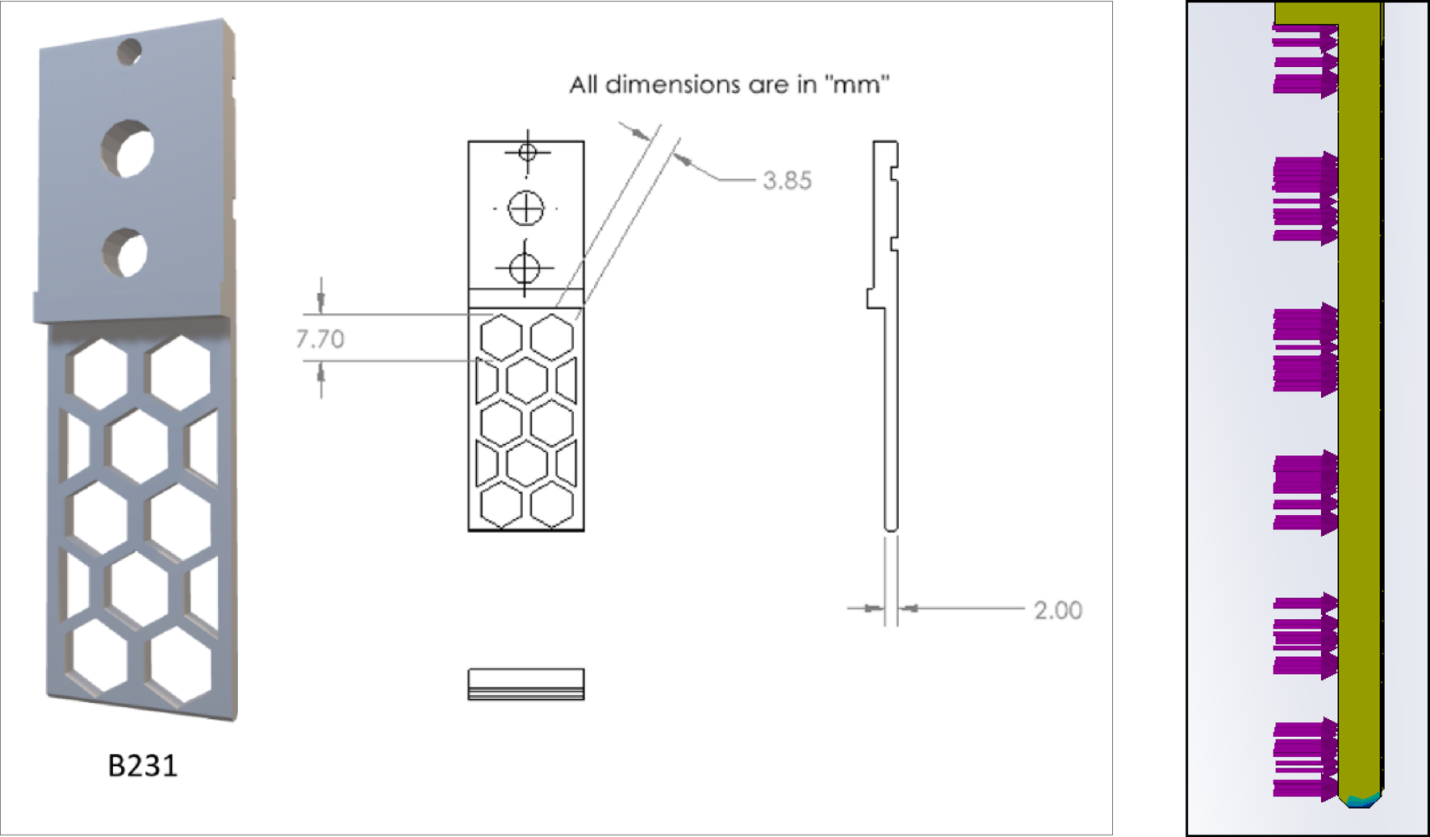
Design and 3D view of V13-B231 (left) and topology study of V13-B231 (right).

As metal printing is stronger than traditional 3D printing, the finger thickness (w) can be reduced, thus increasing the inclination angle and granular flow. Figures 17 and 18 show the design and topology study of the reduced thicknesses of the previous models, V13-A12 and V13-B23. The final model numbers are V13-A121 and V13-B231.

In Table 3 shows the comparison, indicating how much the angle has changed of the updated and final version of the designs (V13-A121 and V13-B231).

**Table 3:** Comparison of the designed model.

Sl. no	Design number	Finger width (w) (mm)	Dia or length of the unit hole (mm)	Total area (mm × mm)	Free area (mm × mm)	Ratio %	The natural inclination of the wall (degree)
A	B	C	D	E	F	G = F/E	H
1	V13-A12	4	7	760	384.85	50.64	60.26
2	V13-A121	2	7	760	384.85	50.64	**74.1**
3	V13-B23	4	3.5*	760	318.26	41.88	60.25
4	V13-B231	2	3.5*	760	318.26	41.88	**74.1**

*sl no. 3 and 4, column D is the one side length of a hexagonal hole.

Significant values are in bold.

**Figure 19:**
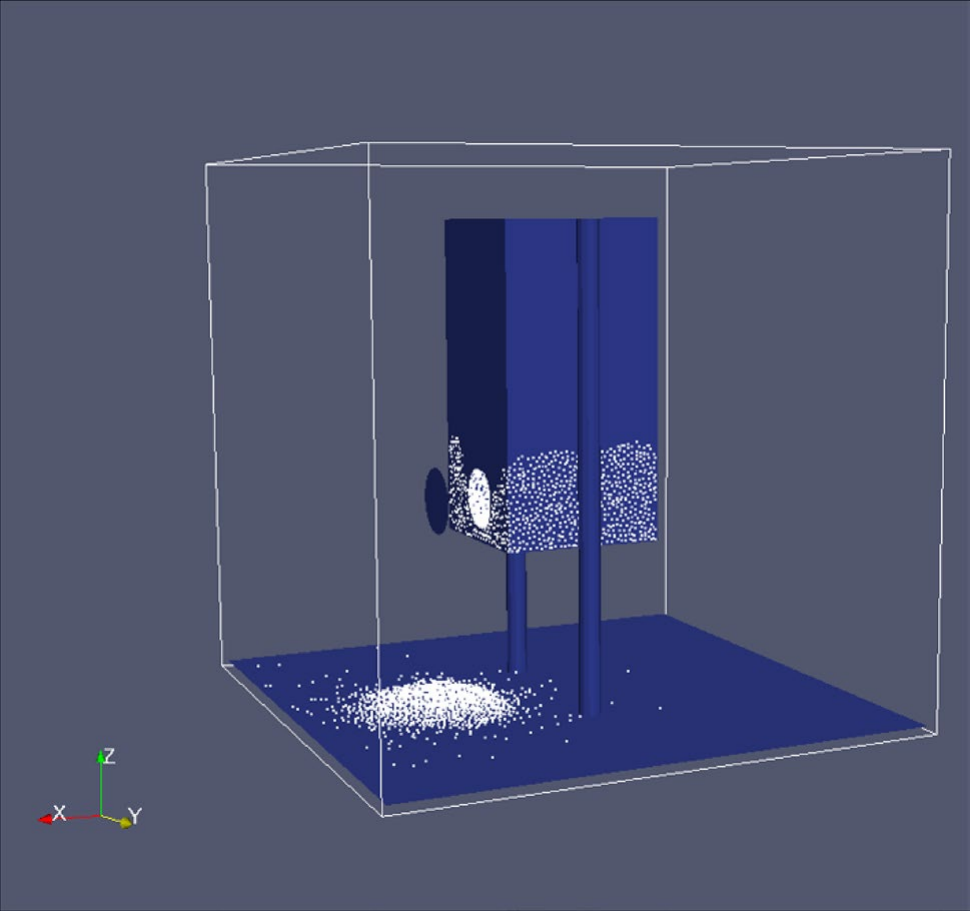
DEM with LIGGGHTS and PARAVIEW.

To examine numerically the flowability of circular and hexagonal holes, a simulation analysis was performed using the Discrete Element Method (DEM). In DEM, granular materials are simulated computationally. Using this method, individual particles are modeled as discrete entities, and their interactions are simulated over time. Various forces are considered in DEM when modeling the interactions between particles, including gravitational forces, frictional forces, and contact forces. Figure 19 shows a setup developed with LIGGGHTS and PARAVIEW software to test the mass flow rate over time.

In this stage, a 3D model is built using SolidWorks software, which is then transformed into a STL file for LIGGGHTS input. PARAVIEW is used to visualize the simulation after it has been completed.

**Figure 20:**
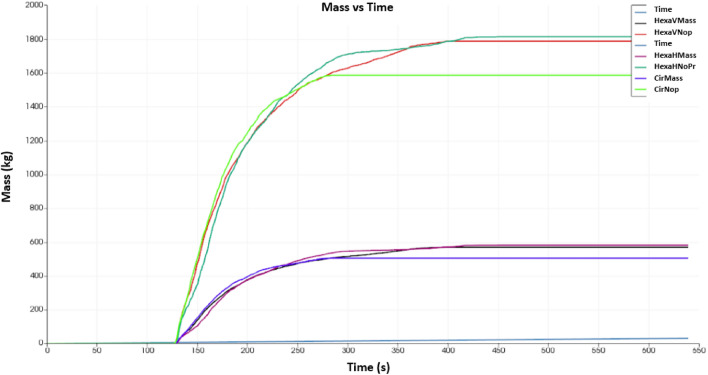
Time vs. mass flow rate.

As shown in Fig. 20, the circular shapes have a higher flowability at the beginning. In a steady state, hexagonal-shaped holes offer higher flowability. The hexagonal shape shows a flow rate of approximately 600 kg per second, while the circular one indicates roughly 500 kg per second. Two orientations (vertical and horizontal) of hexagonal shapes show similar mass flowability results. This means that design number V13-B231 will be more effective than design number V13-A121.

## Conclusion and future work

An additive manufacturing process with robotic grippers can be automated, precise, versatile, and efficient, improving quality and productivity. However, it was difficult to pick up some objects when most of the sample remained under granular particle conditions. This problem has been solved by adding in the existing gripper to reduce the drag force and increase particle flowability through the finger to reduce contact force. The design study started with a blind design (Model V11). There were three designs from the V11, namely V12, V13, and V14. Using a preliminary force simulation, V13 has been further modified into seven models, of which two use hexagonal holes instead of circular ones. Table 1 compares these seven models with the V11. According to the data, model V13-A11 has the maximum free surface compared to other circular models, while model V13-B22 has the maximum free surface for hexagonal models. Additionally, these two models have good inclination angles, another factor that controls granular flow.

Based on the topology study, these two models further examine the possibility of thickness reduction, which increases the inclination angle. However, the topology study did not indicate any possibility of material reduction. As well as this, these two models require a slight modification to reduce the corner sharpness for V13-A11 and V13-B23, which decreases the number of hexagonal shapes by increasing the length of the sides, 's'. Table 2 shows that V13-A12 has the same factors as V13-A11, but V13-B23 has an increased free surface and degree of angle. Moreover, these two modified fingers have been printed using a 3D printer to verify their compatibility with the experimental setup. The experiment results reveal a new problem: bending when picked up from specific locations. It was reprinted with a metal 3D printer, which was coated to reduce surface roughness. The new experiment shows the effectiveness of metal printing, which further motivates reducing the design's thickness. Therefore, V13-A121 and V13-B231 are the final designs of the study, which also show an increase in inclination angle (Table 3). According to the numerical analysis, hexagonal fingers (V13-B231) is more effective than circular fingers due to the higher mass flow of hexagonal holes as compared to circular ones.Based on the literature review findings and our understanding, this study presents an innovative method for designing robotic finger designs using binder jet additive manufacturing. The outcomes of this research are as follows.

- A novel approach to design has been developed for an autonomous binder jet additive manufacturing pick-up system.
- For specific types of samples, two novel designs have been developed to reduce the contact force required to pick up objects from the manufacturing 3D models.
- For specific types of samples, two novel designs have been developed to increase the flowability to pick up objects from the manufacturing 3D models.
- Numerical analysis has shown that hexagonal free areas are more effective for picking objects in granular media than circular free areas.

This study has some limitations, as do most research studies. Only four specific types and specific materials have been considered for the design and analyses and results might be different for the different materials and samples. In the future, this study will continue considering different shapes and materials. In addition, this study did not measure the flowability coefficient. In the future, this calculation will be done with numerical analysis using the discrete element method (DEM).

## Data Availability

The published article contains all data generated or analyzed during this study. The corresponding author may also be able to provide data upon request.
